# VEP-based acuity estimation: unaffected by translucency of contralateral occlusion

**DOI:** 10.1007/s10633-021-09840-0

**Published:** 2021-05-11

**Authors:** Sven P. Heinrich, Isabell Strübin, Michael Bach

**Affiliations:** 1 Eye Center, University of Freiburg Medical Center, Killianstr. 5, 79106 Freiburg, Germany; 2 Faculty of Medicine, University of Freiburg, Freiburg, Germany

**Keywords:** Visual acuity, Visual evoked potential, Binocular interaction, Occlusion, Rivalry

## Abstract

**Purpose:**

Visual evoked potential (VEP) recordings for objective visual acuity estimates are typically obtained monocularly with the contralateral eye occluded. Psychophysical studies suggest that the translucency of the occluder has only a minimal effect on the outcome of an acuity test. However, there is literature evidence for the VEP being susceptible to the type of occlusion. The present study assessed whether this has an impact on VEP-based estimates of visual acuity.

**Methods:**

We obtained VEP-based acuity estimates with opaque, non-translucent occlusion of the contralateral eye, and with translucent occlusion that lets most of the light pass while abolishing the perception of any stimulus structure. The tested eye was measured with normal and artificially degraded vision, resulting in a total of 4 experimental conditions. Two different algorithms, a stepwise heuristic and a machine learning approach, were used to derive acuity from the VEP tuning curve.

**Results:**

With normal vision, translucent occlusion resulted in slight, yet statistically significant better acuity estimates when analyzed with the heuristic algorithm (*p* = 0.014). The effect was small (mean ΔlogMAR = 0.06), not present in some participants, and without practical relevance. It was absent with the machine learning approach. With degraded vision, the difference was tiny and not statistically significant.

**Conclusion:**

The type of occlusion for the contralateral eye does not substantially affect the outcome of VEP-based acuity estimation.

## Introduction

Visual evoked potentials (VEPs) are frequently used to obtain an objective acuity estimate in cases where psychophysical acuity testing is considered unreliable [1]. Although there are different variants of the method (e.g., [2–4]), the basic concept is straightforward. Stimulus patterns, e.g., checkerboard patterns, of different coarseness are presented. If the stimuli are resolved, a VEP response is evoked, and if there is no VEP response recorded, it is assumed that the pattern could not be resolved, i.e., that its coarseness is below the patient’s resolution threshold.

Typically, VEP recordings are performed monocularly. However, it is long known that binocular interactions may occur during monocular psychophysical testing. For instance, Wildsoet et al. [5] found effects on high- and low-contrast acuity and contrast sensitivity. With a translucent occluder, acuity was better than with an opaque occluder, albeit only by a small logMAR difference of 0.02. Kravkov [6] and Tamura [7] both compared conditions with and without the exposure of the contralateral eye to an additional light source and found an acuity effect that depended on the contrast polarity of the test stimulus, in contrast to Hartmann [8] who found an increase in acuity for both polarities. Capris et al. [9] found that monocular sensitivity in perimetry is 0.7 dB higher with a translucent occluder than with an opaque one. Several mechanisms need to be considered when predicting the effect of translucent versus non-translucent contralateral occlusion on VEP-based acuity estimates.

Light falling into the contralateral eye will affect the study eye’s pupil through a consensual pupil reaction, reducing its diameter. This could result in increased acuity due to the effect on the depth of focus and due to reduced optical aberrations [10–12]. On the other hand, previous findings imply that less light falling into the tested eye may result in reduced acuity [13], and with very small pupils (typically below approximately 2–3 mm [14]) diffraction may become relevant [15]. It depends on the actual pupil size and luminance level whether the one or the other effect of a pupil size difference dominates [16, 17]. Furthermore, it seems plausible that the effect of pupil size could also depend on the specific eye disease, and the interaction between eyes might be affected by neuroophthalmological conditions, such as the presence of a relative afferent pupillary defect.

Besides acuity effects, the VEP itself could be affected by a pupil size-related change in retinal illuminance. However, at least with above-threshold stimuli, no sizable effect of pupil size on the VEP was reported [18], consistent with other studies that found no significant dependence of VEP amplitudes on retinal illuminance [19]. However, this does not rule out some effects near the resolution threshold.

It is also conceivable that cortical activation due to the continuous luminance stimulation of the contralateral eye through the translucent occluder may interact with the weak activation resulting from the stimulation of the study eye near the perceptual threshold. This may facilitate a VEP response by pushing the activation to exceed a certain neuronal activation threshold, or it might reduce response detectability by increasing the noise level and reducing the signal-to-noise ratio. It is not obvious how much effect the presentation of a homogenous luminance field to the contralateral eye will have, because the retina performs extensive preprocessing of the stimulus information, with the majority of retinal ganglion cells transmitting information about luminance differences rather than absolute luminance [20].

Another factor to consider is binocular rivalry, where incongruent stimuli presented to the left and right eye are processed in a competitive manner, resulting in alternating percepts, which typically switch every few seconds [21, 22]. Already Helmholtz [23] more than 150 years ago discussed the effects of luminance on stimulus visibility in rivalry situations. It is known that increasing stimulus luminance in one eye increases the perceptual dominance of that stimulus [24]. This is compatible with Levelt’s well-known propositions on rivalry in their original and recently updated form [25]. Investigations by Rozhkova et al. [26] provide evidence for an effect of luminance on rivalry in the case of homogenous fields. That study assessed rivalry between dark and bright fields and found that the bright field typically dominated perception, while the dark field was only perceived for short episodes. In other words, dark and bright homogenous fields have different potencies in dominating the percept. In the present experiment, this means that translucent contralateral occlusion and non-translucent contralateral occlusion are likely to differ in their ability to induce competition with the pattern stimulus that is presented to the tested eye.

There are several studies that imply a susceptibility of the VEP to rivalry. For instance, rivalry has been proposed to underlie amplitude fluctuations in monocularly recorded VEPs [27] and in VEPs that were recorded to the stimulation of one eye, while the other eye received competing stimulation [28]. Tyler and Apkarian [29] found that the binocular VEP depends strongly on the relative orientation of the grating stimuli that were presented to the two eyes. Brown and Norcia [30] used frequency tagging to extract eye-specific responses from the VEP and found the responses of the two eyes to fluctuate antisynchronously and in correlation with the corresponding subjective reports of rivalry. Related to this, continuous flash suppression, where the perception of target stimuli presented to one eye is suppressed by flashing stimuli presented to the other eye, has been shown to strongly modulate steady-state VEP responses to the target stimuli [31].

In summary, there are several mechanisms through which differences in the occlusion of the contralateral eye may affect the VEP that is recorded by stimulating the study eye. The present study addresses the question whether this results in a difference in VEP-based acuity estimates. Due to the design of the acuity estimation algorithm, any change of VEP amplitude by the same percentage over all check sizes will not affect acuity estimates. However, if amplitude effects depend on stimulus coarseness (e.g., check size), acuity estimates are likely to change. For instance, it appears quite plausible that the effects of binocular rivalry are strongest with stimuli near the resolution threshold that are barely visible. The signal-to-noise ratio of a near-threshold response may even become so small that the response is not significant anymore, which would affect the range of data points that are used to compute the acuity estimate (see Methods section).

## Methods

The present study followed the tenets of the declaration of Helsinki and was part of a series of experiments that had been approved by the local institutional review board. A non-blinded counterbalanced crossover design was chosen.

### Participants

In total, 17 participants (6 males) participated after providing informed consent. All had normal or corrected-to-normal visual acuity. One participant was excluded due to excessive eye blinks, leaving 16 participants that were included in the analysis.

### VEP recording and evaluation

VEP-based acuity estimates were obtained using the procedure described by Bach et al. [32], which has been successfully used in its original or modified version in several studies (e.g., [33–36]). For each experimental condition (i.e., for a single acuity estimate), the recording duration was typically in the order of 5–10 min, depending on the number of eye blinks, which were rejected based on a 120-μV threshold criterion. The procedure was in agreement with the respective ISCEV extended protocol [37]. In short, steady-state VEPs were recorded to six different check sizes. The response at the first harmonic was obtained through Fourier analysis and corrected for noise [38], and the corresponding statistical significances were estimated [39]. This results in a tuning curve which relates the response amplitude to the dominant spatial frequency of the stimulus. An algorithm selects the appropriate data points based on the statistical significance of the response, fits a straight line and determines the abscissa intercept, which represents the “VEP SF limit” as proposed by the ISCEV extended protocol. In the degraded vision condition, the large number of check sizes that were too small to be resolved increased the likelihood of spurious significances (multiple testing problem). To reduce this effect for the purpose of the present study, we manually corrected for this in obvious cases by defining these points as non-significant before the heuristic algorithm was applied.

A conversion factor is applied to the intercept spatial frequency with the aim of making the result numerically comparable to standard subjective decimal acuity values. In our current implementation, which is also used for clinical routine applications, the result is clipped to ≤ 1.6 (logMAR ≥  − 0.2), because larger values, far beyond the range covered by the stimulus check sizes, have a relatively high likelihood of being imprecise, while at the same time a differentiation between decimal acuity values above 1.6 is irrelevant for the typical application of the method in cases of unexplained visual loss.

For comparison, we also estimated acuity using a machine learning approach that we have recently proposed [3]. This was only done for the condition with undegraded acuity and only after the heuristic algorithm revealed an effect of contralateral occlusion (see “Results” section) in order to test whether the effect is specific to the type of analysis after we failed to find no systematic change of the tuning curve. The machine learning approach uses a neural network that has been trained with previous VEP tuning curves and corresponding behavioral acuity data to estimate acuity from new tuning curves. Application of the machine learning approach to the data of the degraded acuity condition would not have resulted in meaningful acuity estimates because the training data set did not include acuity levels in that range.

### Specific study procedure

In each participant, one eye was selected randomly as study eye and used in all experimental conditions. With this eye, VEP-based acuity estimates were obtained either with normal vision or with vision degraded by placing a diffusing filter (1° Light Shaping Diffuser, Luminit, Torrance, CA, USA) in a trial frame in front of the study eye. This filter produced Gaussian blur and reduced acuity to around 0.09 decimal acuity (logMAR = 1.06) as measured behaviorally in a recent study [40].

The fellow eye was either covered with an eye patch as used for amblyopia treatment (ORTOPAD, Trusetal Verbandstoffwerk GmbH, Schloss Holte-Stukenbrock, Germany; light transmission measured to be less than 1%, although transmittance of the adhesive material at the rim of the patch is somewhat higher), or was supplied with a strongly diffusing, albeit translucent occluder made from polymethyl methacrylate with light-diffusing beads embedded throughout the material (PLEXIGLAS Satinice 0D010 DF, Evonik Performance Materials GmbH, Darmstadt, Germany; thickness 3 mm, light transmission 83% as per the data sheet). This occluder was also inserted into the trial frame. Thus, the experiment involved two diffusors, one for occluding the contralateral eye (used alternatingly with the opaque eye patch), which completely nullifies any perception of shape, and one for the study eye (used solely in the degraded acuity condition), which only moderately reduces acuity. Both eyes were also supplied with the appropriate corrective lenses (individual refraction and near addition for the stimulus distance).

The rationale for choosing the two different types of occlusion for the fellow eye is that they represent the extremes of a continuum of light levels to which an occluded eye could be exposed in clinical practice. The case of a non-translucent occluder inserted into a trial frame (allowing straylight to enter from the sides) would be expected to have an effect that is in between these extremes.

The two acuity conditions in the study eye and the two occlusion conditions in the fellow eye resulted in a total of 4 conditions in a 2 × 2 design. The order of conditions was counterbalanced across participants to minimize sequence effects.

All analysis was performed with IGOR Pro 7 and 8 (Wavemetrics, Inc.). Statistical testing (repeated-measures comparison of medians) was performed with a permutation test, and confidence intervals were bootstrapped [41]. Because the machine learning approach was only a secondary analysis, it was not included in the correction of multiple testing. At one occasion, we supplementarily assessed the mean instead of the median, as it better reflected certain characteristics of the data (see Results section) [41].

## Results

**Fig. 1:**
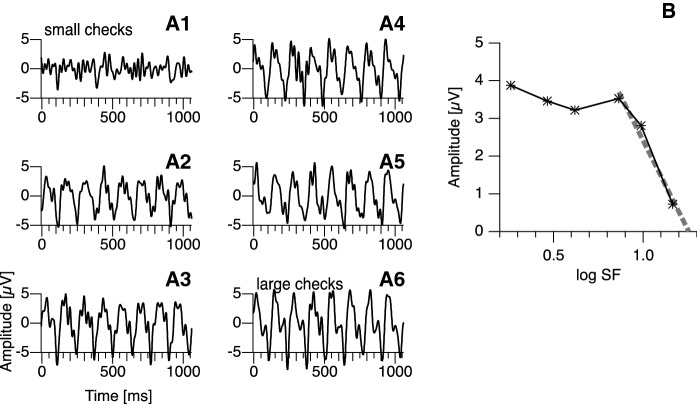
Graphs A1–A6 show typical time-course data (average of 1-s segments) of one participant for all check sizes. Graph B shows the resulting tuning curve, i.e., the amplitude of the first harmonic as a function of the logarithm of the stimulus’ spatial frequency. Asterisk-shaped markers indicate that the response is significantly different from noise (single-test *α* = 0.05). The dashed line was fitted to the descending slope of the tuning curve following the heuristic algorithm described by Bach et al. [32]. The abscissa intercept is taken as a measure of the resolving power of the visual system, which can be converted into an estimate of visual acuity

**Fig. 2:**
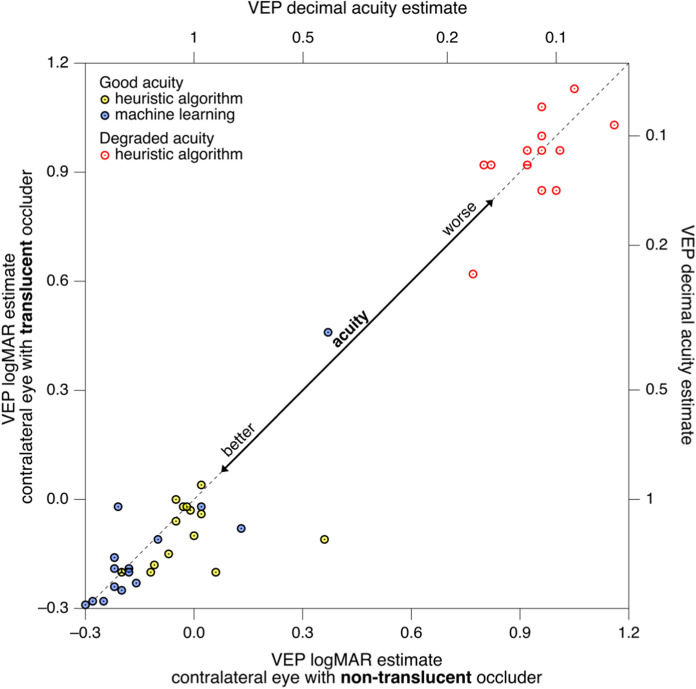
Scatterplots comparing acuity estimates (converted from VEP spatial frequency thresholds) obtained with a translucently occluded occluders (ordinate) to those obtained with a non-translucently occluded contralateral eye (abscissa). In three cases the data points of two or three participants coincide. These are represented as ‘sunflower’ markers [42] with the number of petals (sectors) indicating the number of data points. Results with good and degraded acuity clearly segregate. With the heuristic algorithm, estimated acuity with good acuity tends to be better with non-translucent occlusion than with translucent occlusion. This was not the case with the machine learning approach

Figure 1 shows the averaged time-course data for a sample participant, recorded with degraded acuity. The scatter plot in Fig. 2 displays how VEP estimates depend on the type of occlusion of the contralateral eye. It furthermore shows some discrepancy between the heuristic algorithm and the machine learning approach in particular with degraded vision, irrespective of the type of contralateral occlusion.

### Acuity estimation based on the heuristic algorithm

No acuity estimate could be obtained with degraded vision in two participants. With undegraded vision, the VEP-based estimates suggest slightly better acuity (lower logMAR values) with a translucent occluder (median logMAR difference =  −0.064, CI_95%_ = [−0.0, −0.077]; *p* = 0.030, significant at a single-test level but not with a Bonferroni correction with a factor of 2 at a family-wise α of 0.05). Performing this analysis with respect to the mean instead of the median shows a clear significance not only at a single-test level but also with Bonferroni correction (mean logMAR difference =  −0.064, CI_95%_ = [−0.016, −0.135]; *p* = 0.0024), reflecting the fact that a number of participants showed little or no difference, while most of those who showed a sizable difference had the effect in the same direction. If the most outlying data point is excluded (see Fig. 1), the mean effect was still significant at a single-test level (*p* = 0.027), but not at a family-wise level. No difference was found with degraded acuity (median logMAR difference =  −0.000, CI_95%_ = [0.094, −0.070]; *p* = 1.00).

**Fig. 3:**
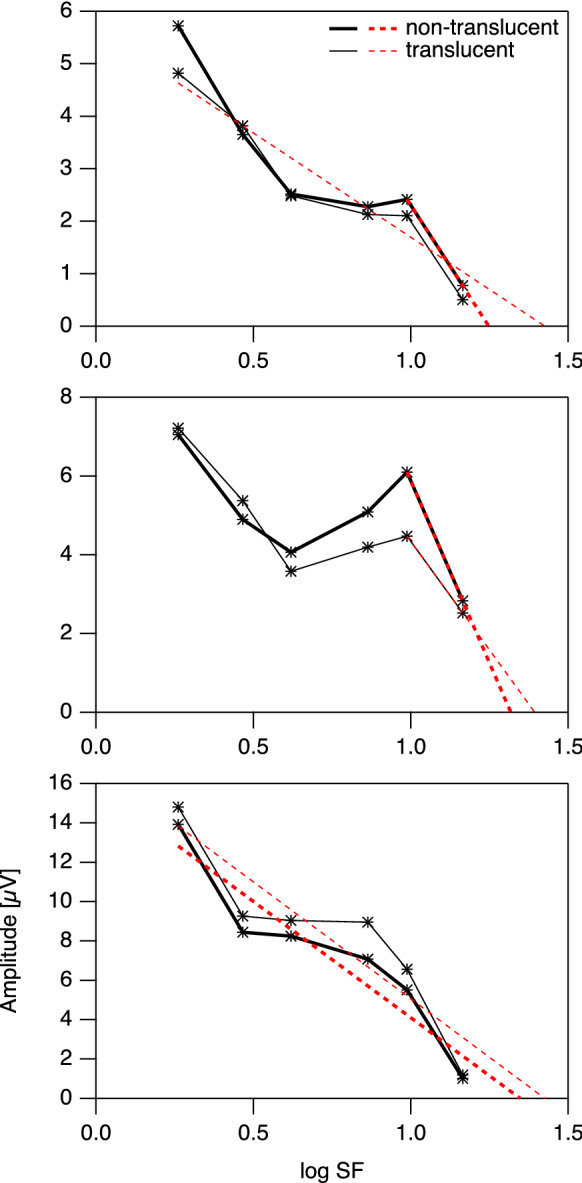
Three examples of relatively similar tuning curves obtained from different individuals with undegraded vision in the tested eye and either non-translucent (bold line) or translucent (thin line) occlusion of the contralateral eye. The dashed straight lines are fitted to the tuning curves in accordance with the heuristic algorithm. Asterisk-shaped markers indicate response significance (see Fig. 1). In all three cases shown, spatial frequency thresholds (abscissa intercept of fitted line) were higher with translucent occlusion. However, the tuning curves show no obvious common pattern that would consistently underlie this effect

In order to better understand the effects of contralateral occlusion type, we visually inspected the respective tuning curves. However, we did not find any obvious pattern in the data points that would consistently explain for most or all affected participants why measurements with translucent occlusion of the contralateral eye would result in better acuity estimates (lower logMAR values). Figure 3 shows for three example participants how the tuning curves differ between conditions.

### Acuity estimation based on machine learning

When applying the machine learning approach to estimate VEP acuities, differences in acuity estimates between types of contralateral occlusion were minimal and not statistically significant (undegraded acuity, median logMAR difference −0.010, CI_95%_ = [−0.022, 0.008]; *p* = 0.71; degraded acuity not assessed, see Methods section).

## Discussion

The present data suggest that translucent occlusion does not have a relevant effect on VEP-based acuity estimates. With the heuristic algorithm (but not with the machine learning approach), translucent occlusion of the contralateral eye is associated with a slightly higher mean VEP-based acuity estimate (lower logMAR values) in the tested eye when vision is undegraded. The effect is not present in several of the participants, and it is not found when vision is degraded. The mean effect with undegraded vision (logMAR difference of 0.06) is larger than that reported by Wildsoet [5] for psychophysical letter-chart acuity (difference of 0.02), although the confidence interval of the former includes the latter.

While the effect is absent when the machine learning approach is used for acuity estimation, it is nevertheless interesting to note that we found no consistent pattern of tuning curve changes that would readily explain why the heuristic algorithm tends to yield different estimates with different types of contralateral occlusion. It is likely that this issue is related to the fact that the tuning curve has no further data points at spatial frequencies higher than the slope region of the curve, as opposed to the condition with degraded acuity. This makes fitting the straight line less robust.

With undegraded vision, acuity estimates from machine learning suggest better acuity (lower logMAR) than estimates from applying the heuristic algorithm (this was not assessed for degraded vision). We compared the acuity estimates to behavioral acuity and found that estimates from machine learning show better agreement. Such a trend can also be identified in our previous study [3] for the case of normal vision. For degraded vision, we did not obtain behavioral acuity estimates in the present study as the focus of the study was a comparison between VEP-based estimates. However, we know from a previous study [40] that the filter used for degradation typically reduces acuity to a logMAR value of about 1.0, which is matched quite well by the acuity estimates obtained with the heuristic algorithm.

From a practical perspective, the present results imply that the translucency of the contralateral occlusion has little relevance for VEP-based acuity estimation. In most cases, the logMAR difference is not larger than 0.1 even with the heuristic algorithm.

## Data Availability

Data are available from the corresponding author upon reasonable request.

## Declarations

### Statement on the welfare of animals

This article does not contain any studies with animals performed by any of the authors.
